# Clinical outcomes with first-line tislelizumab plus lenvatinib in unresectable hepatocellular carcinoma: a retrospective analysis

**DOI:** 10.7717/peerj.20591

**Published:** 2026-01-19

**Authors:** Jizong Lin, Qingxun Zhou, Zhicheng Yao, Qingliang Wang, Shilei Xu, Zhiyong Xiong, Xi Dang, Hao Liang, Bo Liu

**Affiliations:** Department of Hepatobiliary, Spleen and Pancreatic Surgery, The Third Affiliated Hospital of Sun Yat-sen University, Guangzhou, Guangdong, China

**Keywords:** Retrospective analysis, Efficacy, First-line treatment, Hepatocellular carcinoma, Lenvatinib, Tislelizumab

## Abstract

**Purpose:**

This retrospective analysis aimed to assess the therapeutic efficacy and adverse event profile of tislelizumab combined with lenvatinib in patients with unresectable hepatocellular carcinoma (uHCC), predominantly of hepatitis B virus (HBV) etiology. While this combination has shown promise in clinical trials, real-world data in HBV-endemic populations remain sparse.

**Methods:**

A total of 163 uHCC patients who initiated first-line systemic therapy with tislelizumab and lenvatinib between January 2021 and February 2025 were retrospectively analyzed. Treatment response was evaluated according to the modified Response Evaluation Criteria in Solid Tumors (mRECIST). Key clinical endpoints included objective response rate (ORR), overall survival (OS), progression-free survival (PFS), disease control rate (DCR), treatment-emergent adverse events (TRAEs), and other relevant outcomes. Prognostic factors were explored through subgroup analyses and multivariate Cox proportional hazards modeling.

**Results:**

Of the 163 patients (84.7% HBV-related), the ORR was 25.8%, DCR was 67.5%, and median PFS was 13.87 months. Additionally, 16.6% of patients achieved conversion therapy and subsequent surgical resection. Subgroup and multivariate analyses indicated that a larger tumor burden, particularly tumors ≥5 cm, was associated with shorter PFS but did not significantly affect OS. TRAEs were observed in 86% of patients, with grade ≥3 events occurring in 5%.

**Conclusions:**

This analysis supports that tislelizumab plus lenvatinib provides substantial clinical benefit in HBV-related uHCC, including potential for conversion to surgical resection. Tumor burden emerged as a key predictor of progression. The regimen demonstrated a favorable safety profile, reinforcing its potential as a frontline treatment in HBV-endemic areas.

## Introduction

As one of the most prevalent primary liver cancers, hepatocellular carcinoma (HCC) represents a significant global health burden, accounting for approximately 4.3% of newly diagnosed cancers and 7.8% of cancer-related deaths worldwide (Bray et al., 2024; Wang et al., 2024a). In China, HCC remains highly prevalent and fatal, ranking among the top five cancers in terms of both incidence and mortality (Zheng et al., 2024; Zheng et al., 2023).

While curative approaches such as resection and transplantation offer favorable outcomes in early-stage HCC, the majority of patients are diagnosed at advanced stages, making systemic therapy the cornerstone of treatment in clinical practice (Llovet et al., 2018; Yang et al., 2023). Tyrosine kinase inhibitors (TKIs) are commonly used in the first-line treatment of unresectable HCC (uHCC), but their efficacy remains suboptimal, as evidenced by objective response rates (ORRs) ranging from 2.7% to 24.1% (Kudo et al., 2018; Llovet et al., 2008; Qin et al., 2021).

Immune checkpoint inhibitors (ICIs) have emerged as promising therapeutic agents in cancer treatment. However, monotherapies like pembrolizumab (Finn et al., 2020c) and nivolumab (Yau et al., 2022) have failed to demonstrate significant survival benefits over sorafenib. In contrast, tislelizumab, a selective anti-programmed cell death protein-1 (PD-1) antibody, has shown a 14.3% ORR and non-inferior overall survival (OS) compared to sorafenib in Phase II and III trials (Qin et al., 2023b; Ren et al., 2023).

Given that TKIs can enhance tumor immunogenicity by modulating the tumor microenvironment through inhibition of vascular endothelial growth factor (VEGF) and angiopoietin-2 (ANG2), combining TKIs with ICIs represents a rational strategy to improve treatment efficacy (Fukumura et al., 2018; Huinen et al., 2021). Clinical studies have demonstrated improved ORRs and survival outcomes with combinations such as lenvatinib plus pembrolizumab (Finn et al., 2020a; Llovet et al., 2023) and camrelizumab plus rivoceranib (Qin et al., 2023a), compared to monotherapy regimens. Notably, the combination of tislelizumab and lenvatinib has also shown promising efficacy, with ORRs exceeding 40% in recent trials (Xu et al., 2024).

Tislelizumab, an engineered anti-PD-1 monoclonal antibody, features an optimized Fc segment that minimizes binding to Fc gamma receptors (Fc*γ*R), reducing antibody-dependent phagocytosis. This mechanism may help mitigate resistance within the immunosuppressive tumor microenvironment often associated with hepatitis B virus (HBV)-related HCC, thereby enhancing antitumor activity (Zhang et al., 2018; Zhang et al., 2024b). Lenvatinib not only exerts anti-angiogenic effects but may also facilitate immune cell infiltration, acting synergistically with PD-1 blockade, which provides a strong rationale for exploring this specific combination.

However, most existing evidence originates from randomized controlled trials (RCTs), which typically involve selected patient populations under tightly controlled conditions, limiting their applicability to real-world clinical settings. This study provides real-world evidence on the therapeutic efficacy and adverse event (AE) profile of combining tislelizumab with lenvatinib, particularly in a Chinese population with HBV-driven HCC.

## Materials and Methods

### Study population

**Figure 1:**
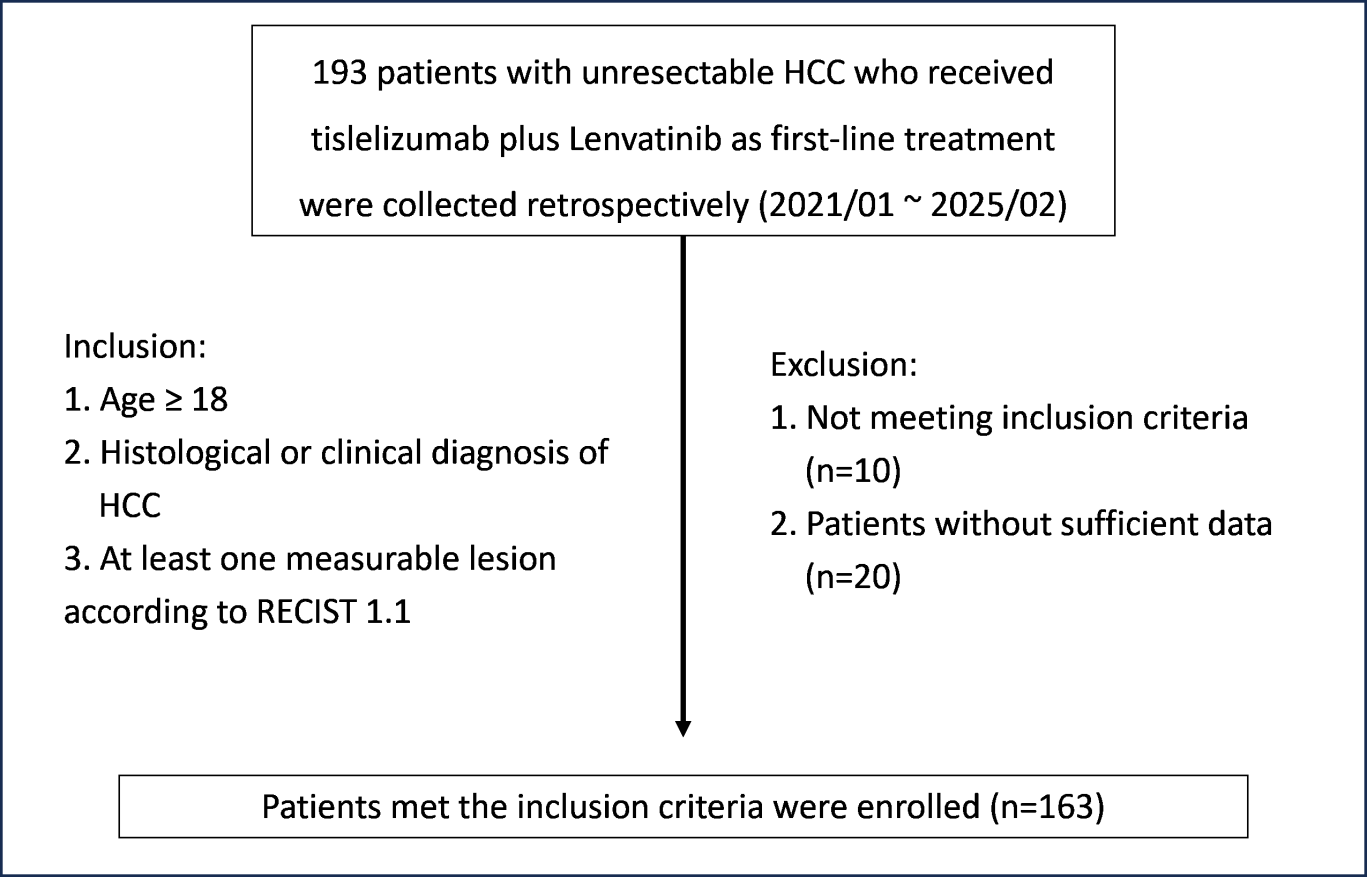
Flowchart of patient selection. Abbreviations: HCC, hepatocellular carcinoma; RECIST, Response Evaluation Criteria in Solid Tumors.

This study was approved by the Ethics Committee of the Third Affiliated Hospital of Sun Yat-sen University (No. II2025-291-01), with a waiver of informed consent due to its retrospective design. A total of 163 patients diagnosed with uHCC who began first-line therapy with tislelizumab and lenvatinib between January 2021 and February 2025 were included in the analysis. Eligibility criteria required participants to be 18 years or older, with a confirmed diagnosis of HCC through histological or clinical evaluation, and at least one measurable lesion as per the Response Evaluation Criteria in Solid Tumors (RECIST), version 1.1. The patient selection process is depicted in Fig. 1. Thirty patients were excluded: 10 due to loss to follow-up after treatment, resulting in unavailable post-treatment lesion measurements, and 20 due to incomplete laboratory data or lack of complication follow-up information. HCC diagnosis was confirmed through histopathological analysis or imaging consistent with internationally recognized diagnostic criteria. Data collected included tumor size, number of lesions, liver function (Child-Pugh class), alpha-fetoprotein (AFP) levels, presence of portal vein tumor thrombus (PVTT), extrahepatic metastases, and other relevant clinical indicators.

### Treatment procedures

Patients received intravenous tislelizumab at a dose of 200 mg every three weeks, following the standard protocol. Lenvatinib was administered orally daily, with the dose adjusted based on body weight—eight mg for those weighing less than 60 kg, and 12 mg for those weighing 60 kg or more.

### Criteria for conversion therapy and surgical resection

Patients who achieved a response or stable disease after combination therapy were evaluated by a multidisciplinary team (MDT) for potential surgical resection, including consideration for microscopically margin-negative (R0) resection and the patient’s liver function tolerance.

### Outcomes and clinical assessments

Treatment efficacy was assessed using contrast-enhanced computed tomography (CT) or magnetic resonance imaging (MRI), in accordance with the modified RECIST (mRECIST) criteria. Key endpoints included ORR, disease control rate (DCR), OS, and progression-free survival (PFS). PFS was defined as the time from treatment initiation to disease progression or death from any cause, while OS was measured from the start of treatment to death or the most recent follow-up. All AEs occurring during the treatment were recorded and classified according to version 5.0 of the Common Terminology Criteria for Adverse Events (CTCAE) issued by the National Cancer Institute.

### Statistical analysis

PFS and OS were estimated using the Kaplan–Meier method. Baseline demographic and clinical characteristics were summarized using descriptive statistics. Prognostic factors for PFS and OS were identified through univariate and multivariate Cox proportional hazards analyses. In cases with missing laboratory data, multiple imputation techniques were applied. The sample size, determined by the availability of retrospective data, represents one of the largest real-world cohorts for this treatment regimen, providing sufficient statistical power for exploratory analysis of the primary endpoints. Subgroup variables included age, gender, Child-Pugh class, AFP level, presence of PVTT, extrahepatic metastases, and other clinically relevant factors. Statistical analyses were conducted using SPSS (version 26.0; IBM Corp., Armonk, NY, USA) and GraphPad Prism (version 9.5).

## Results

### Patient characteristics

This study included 163 patients with uHCC, with a median age of 54 years. The majority of patients were male (90.8%) and diagnosed with HBV-related HCC (84.7%), consistent with the typical clinical profile of liver cancer in China. Most patients had preserved liver function, with 82.8% classified as Child-Pugh A. Additionally, 65.0% had tumors ≥five cm in size, and 69.9% had multiple lesions. PVTT was present in 37.4% of cases, and 13.5% had extrahepatic metastases. Local liver-directed therapies, such as TACE, HAIC, and ablation, were administered to 89.0% of patients, and 16.6% underwent surgical resection following conversion therapy (Table 1).

**Table 1:** Baseline characteristics of the patients with unresectable HCC (*N* = 163).

**Characteristics**	**Overall, n (%)**
**Median age, years (range)**	54 (18–77)
**Age, years**	
<60	110 (67.5)
≥60	53 (32.5)
**Gender**	
Female	15 (9.2)
Male	148 (90.8)
**Aetiology**	
HBV	138 (84.7)
HCV	7 (4.3)
Others	18 (11.0)
**Child–Pugh**	
A	135 (82.8)
B	28 (17.2)
**Portal hypertension**	
No	79 (48.5)
Yes	84 (51.5)
**AFP**	
<400 ng/mL	105 (64.4)
≥400 ng/mL	58 (35.6)
**Tumor size**	
<5 cm	57 (35.0)
≥5 cm	106 (65.0)
**Tumor number**	
Single	49 (30.1)
Multiple	114 (69.9)
**PVTT**	
No	102 (62.6)
Yes	61 (37.4)
**Extrahepatic metastasis**	
No	141 (86.5)
Yes	22 (13.5)
**Liver local therapy**	
No	18 (11.0)
Yes	145 (89.0)
**Surgical removal after conversion therapy**	
No	136 (83.4)
Yes	27 (16.6)

**Notes.**

Abbreviations AFP alpha-fetoprotein HBV hepatitis B virus HCV hepatitis C virus PVTT portal vein tumor thrombus

### Efficacy of tislelizumab plus lenvatinib

**Figure 2:**
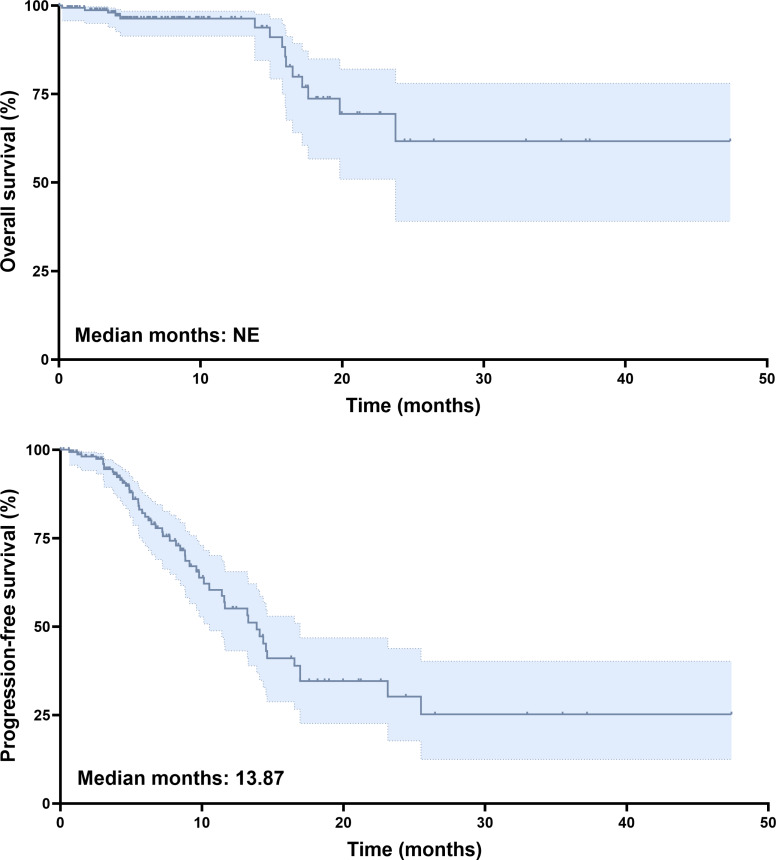
Survival analysis of unresectable HCC patients treated with tislelizumab and lenvatinib. (A) Kaplan–Meier curves of OS. (B) Kaplan–Meier curves of PFS.

The data cutoff for this analysis was June 2025, and the median follow-up duration for the entire cohort was 9.9 months. At the time of analysis, 90% of patients were alive. Tumor response was assessed using mRECIST criteria. Among those treated with tislelizumab plus lenvatinib, two patients (1.2%) achieved complete response (CR), and 40 patients (24.5%) had partial response (PR). Stable disease (SD) was observed in 68 patients (41.7%), while 53 patients (32.5%) had progressive disease (PD). The ORR was 25.8%, and the DCR was 67.5% (Table 2). Kaplan–Meier survival analysis revealed a median PFS of 13.87 months (Fig. 2B). The median OS had not been reached by the time of analysis (Fig. 2A), indicating a favorable long-term survival trend in the study cohort.

**Table 2:** Tumor response after treatment in patients (*N* = 163).

**mRECIST**	**N (%)**
CR	2 (1.2)
PR	40 (24.5)
SD	68 (41.7)
PD	53 (32.5)
ORR (CR/PR)	42 (25.8)
DCR (CR/PR/SD)	110 (67.5)

**Notes.**

Abbreviations CR complete response DCR disease control rate mRECIST modified RECIST ORR objective response rate PD progressive disease PR partial response SD stable disease

### Baseline characteristics and outcomes of patients undergoing conversion surgery

Following conversion therapy, 27 patients (16.6%) underwent successful surgical resection. The baseline characteristics of the resection and non-resection groups are summarized in Table 3. Although the median age was 54 years in both groups, the resection group had a higher proportion of patients aged <60 years (74.1% *vs.* 66.2%). The resection group also demonstrated better liver function and more favorable tumor characteristics: 88.9% of the resection group were classified as Child-Pugh class A compared to 81.6% in the non-resection group. Additionally, a higher proportion of patients in the resection group had tumors <five cm (44.4% *vs.* 33.1%), a single tumor (40.7% *vs.* 27.9%), and no PVTT (70.4% *vs.* 61.0%). Postoperatively, all patients achieved a major pathological response, with no perioperative deaths. All surgical margins were R0, and the median recurrence-free survival (RFS) was 23.67 months.

**Table 3:** Patient baseline characteristics by resection status.

**Characteristics**	**Resection group (*N* = 27), n (%)**	**Non-resection group (*N* = 136), n (%)**
**Median age, years (range)**	54 (26–73)	54 (18–77)
**Age, years**		
<60	20 (74.1)	90 (66.2)
≥60	7 (25.9)	46 (33.8)
**Gender**		
Female	3 (11.1)	12 (8.8)
Male	24 (88.9)	124 (91.2)
**Aetiology**		
HBV	24 (88.9)	114 (83.8)
HCV	2 (7.4)	5 (3.7)
Others	1 (3.7)	17 (12.5)
**Child–Pugh**		
A	24 (88.9)	111 (81.6)
B	3 (11.1)	25 (18.4)
**Portal hypertension**		
No	17 (63.0)	62 (45.6)
Yes	10 (37.0)	74 (54.4)
**AFP**		
<400 ng/mL	15 (55.6)	90 (66.2)
≥400 ng/mL	12 (44.4)	46 (33.8)
**Tumor size**		
<5 cm	12 (44.4)	45 (33.1)
≥5 cm	15 (55.6)	91 (66.9)
**Tumor number**		
Single	11 (40.7)	38 (27.9)
Multiple	16 (59.3)	98 (72.1)
**PVTT**		
No	19 (70.4)	83 (61.0)
Yes	8 (29.6)	53 (39.0)
**Extrahepatic metastasis**		
No	23 (85.2)	118 (86.8)
Yes	4 (14.8)	18 (13.2)
**Liver local therapy**		
No	6 (22.2)	12 (8.8)
Yes	21 (77.8)	124 (91.2)

**Notes.**

Abbreviations AFP alpha-fetoprotein HBV hepatitis B virus HCV hepatitis C virus PVTT portal vein tumor thrombus

### Cox regression analyses of OS and PFS among patients treated with tislelizumab and lenvatinib

**Figure 3:**
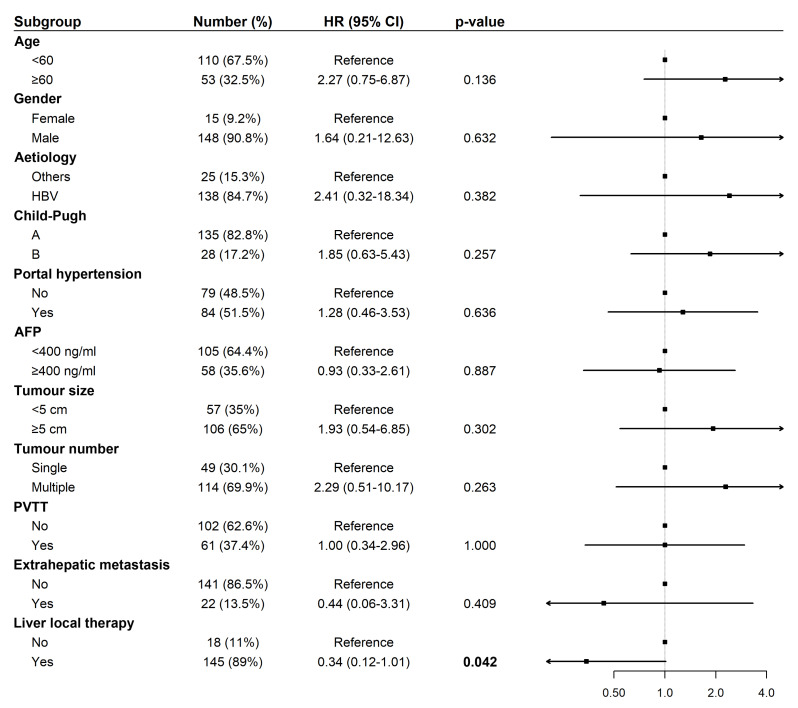
Forest plot of univariate analysis for overall survival (*N* = 163). Abbreviations: AFP, alpha-fetoprotein; HBV, hepatitis B virus; HR, hazard ratio; PVTT, portal vein tumor thrombus.

**Figure 4:**
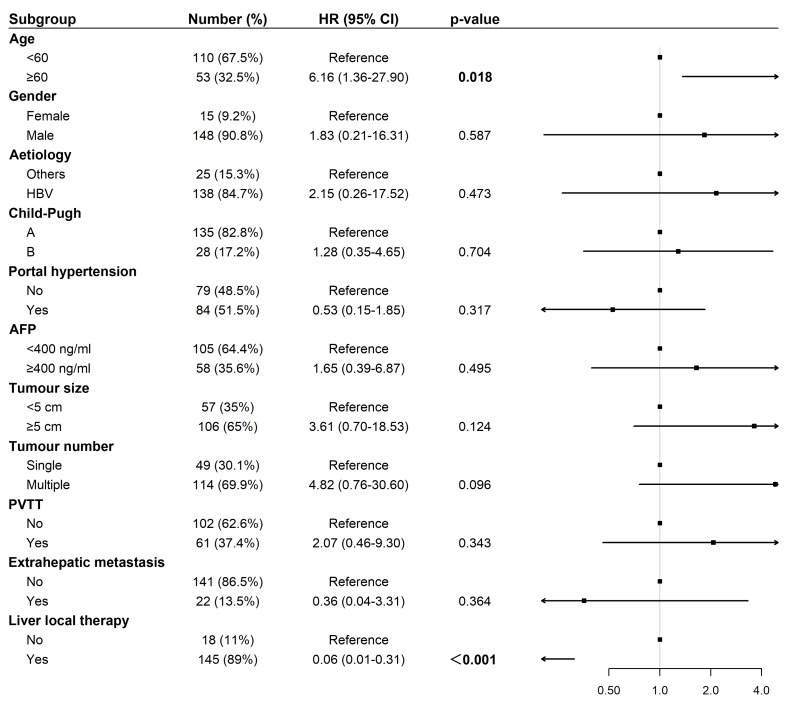
Forest plot of multivariate analysis for overall survival (*N* = 163). Abbreviations: AFP, alpha-fetoprotein; HBV, hepatitis B virus; HR, hazard ratio; PVTT, portal vein tumor thrombus.

Univariate Cox regression analysis for OS revealed that liver-directed therapy was associated with a statistically significant trend toward prolonged survival (Hazard Ratio (HR): 0.34, 95% Confidence Interval (CI) [0.12–1.01], *p* = 0.042; Fig. 3). Multivariate analysis confirmed liver-directed therapy as a significant independent protective factor for OS (HR: 0.06, 95% CI [0.01–0.31], *p* < 0.001). In contrast, age ≥ 60 years was identified as a significant independent risk factor for poorer OS (HR: 6.16, 95% CI [1.36–27.90], *p* = 0.018; Fig. 4).

**Figure 5:**
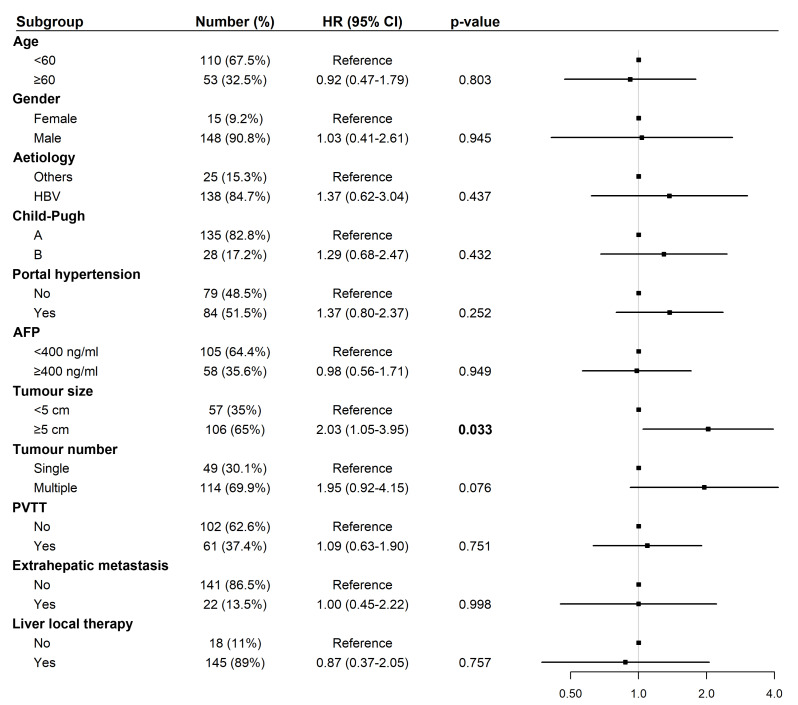
Forest plot of univariate analysis for progression-free survival (*N* = 163). Abbreviations: AFP, alpha-fetoprotein; HBV, hepatitis B virus; HR, hazard ratio; PVTT, portal vein tumor thrombus.

**Figure 6:**
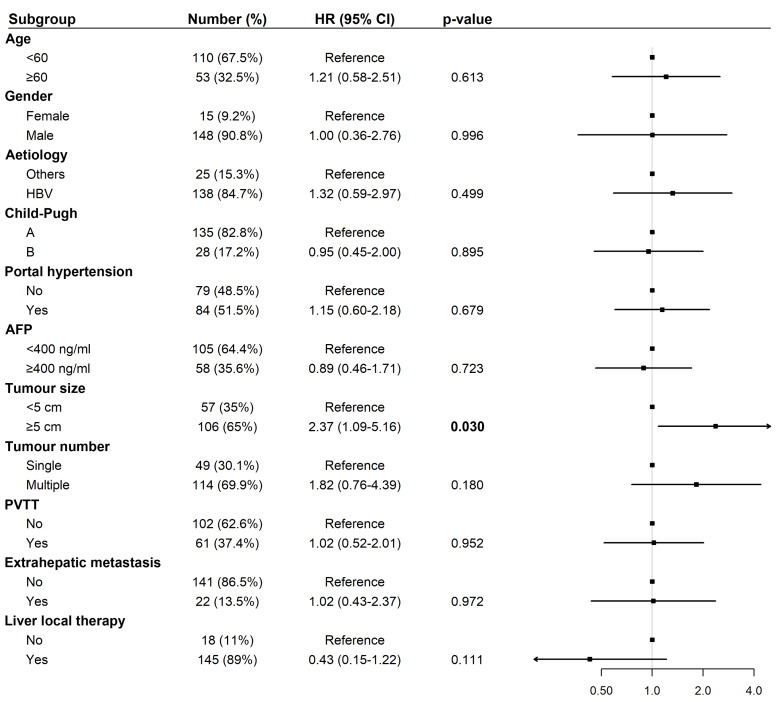
Forest plot of multivariate analysis for progression-free survival (*N* = 163). Abbreviations: AFP, alpha-fetoprotein; HBV, hepatitis B virus; HR, hazard ratio; PVTT, portal vein tumor thrombus.

For PFS, univariate analysis identified tumor size ≥five cm (HR: 2.03, 95% CI [1.05–3.95], *p* = 0.033) as a significant risk factor (Fig. 5). In multivariate analysis, tumor size ≥ five cm remained an independent predictor of shorter PFS (HR: 2.37, 95% CI [1.09–5.16], *p* = 0.030; Fig. 6).

### Correlation of tumor burden with survival parameters

**Figure 7:**
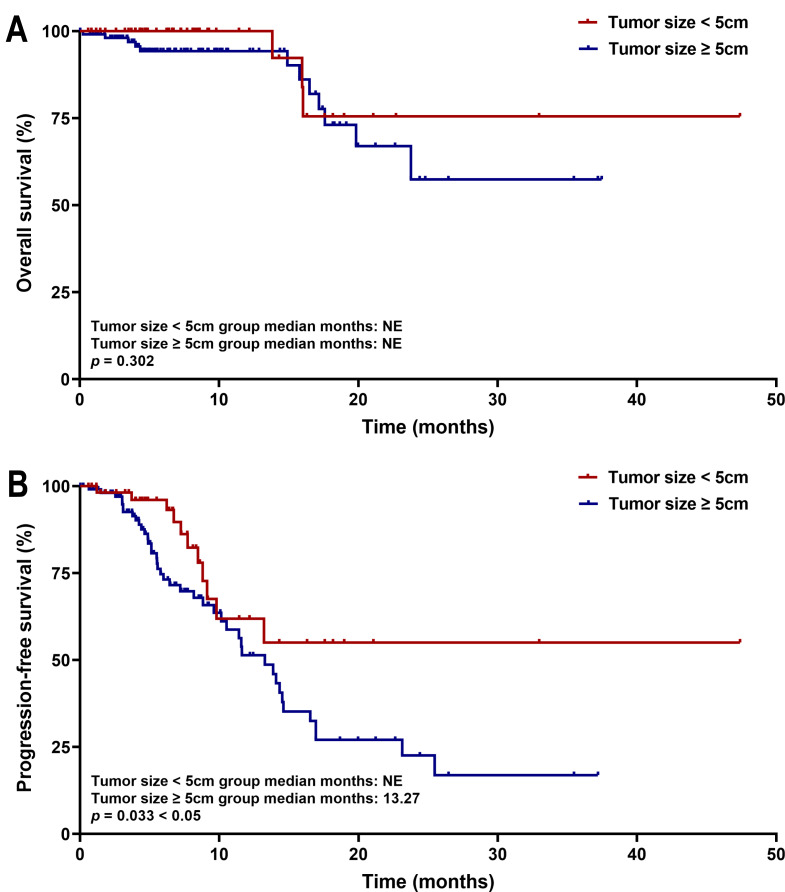
Kaplan–Meier curves of (A) OS and (B) PFS for patients in the tumor size < five cm group and Tumor size ≥ five cm group.

**Figure 8:**
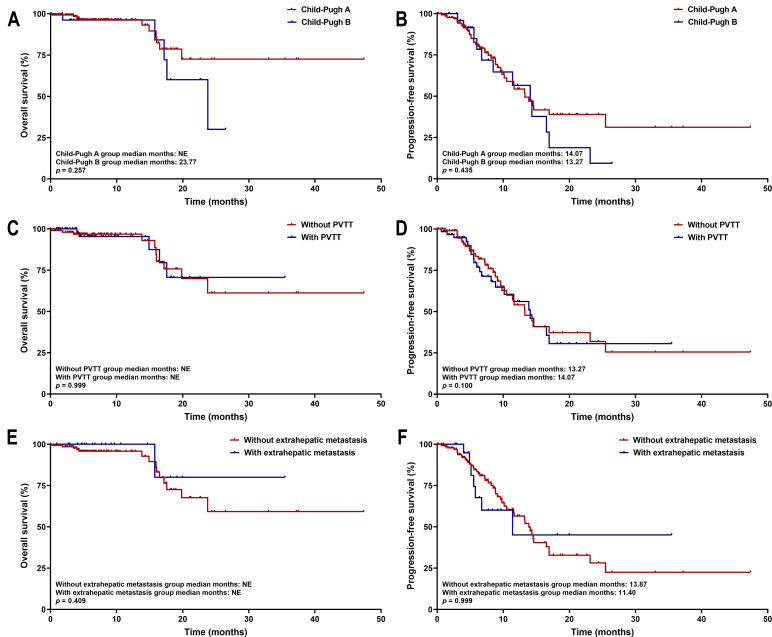
Kaplan–Meier curves of OS and PFS for high-risk subgroups. The analysis is stratified by (A, B) Child-Pugh class, (C, D) PVTT status, and (E, F) extrahepatic metastasis. Abbreviations: PVTT, portal vein tumor thrombus.

Kaplan–Meier survival analysis showed that patients with tumors ≥five cm had significantly shorter PFS than those with tumors <five cm (median PFS: 13.27 months *vs.* not estimable; *p* = 0.033). No significant difference in OS was observed between the two cohorts (both medians not reached, *p* = 0.302) (Fig. 7). These survival trends align with Cox regression results, indicating that larger tumor size is associated with earlier disease progression, but does not significantly affect OS within the current follow-up period. Subgroup survival analysis of other high-risk factors, such as tumor number, PVTT, and extrahepatic metastasis, revealed no significant differences (Fig. 8).

### Safety of tislelizumab plus lenvatinib

A total of 86% of patients experienced at least one treatment-emergent AE (TRAE), with grade 3–4 events occurring in only 5%. The most common adverse reactions of any grade were elevations in aspartate aminotransferase and alanine aminotransferase (both 33%), blood alkaline phosphatase (31%), and blood amylase (29%). Other frequently reported TRAEs included hypertension (25%), increased bilirubin (24%), increased creatinine (23%), and proteinuria (20%). Grade 3–4 events were rare, with no single event exceeding a 2% incidence, except for liver enzyme elevations (Table 4). Only a small number of patients required a dose reduction of lenvatinib due to AEs, and no patients discontinued lenvatinib due to TRAEs.

**Table 4:** Treatment-related adverse events of patients.

**Events**	**All grades**	**Grade 3–4**
Patients with ≥ 1 treatment-related adverse event, n (%)	140 (86)	8 (5)
Aspartate aminotransferase increased	53 (33)	4 (2)
Alanine aminotransferase increased	53 (33)	4 (2)
Blood alkaline phosphatase increased	51 (31)	0 (0)
Blood amylase increased	48 (29)	2 (1)
Gamma-glutamyl transferase increased	42 (26)	0 (0)
Hypertension	40 (25)	0 (0)
Blood bilirubin increased	39 (24)	0 (0)
Blood creatinine increased	38 (23)	0 (0)
Proteinuria	32 (20)	0 (0)
Skin adverse reaction	27 (17)	0 (0)
Platelet count decreased	23 (14)	0 (0)
Hypothyroidism	20 (12)	0 (0)
Diarrhea	19 (12)	0 (0)
Pyrexia	14 (9)	0 (0)
Vomiting	13 (8)	0 (0)
Asthenia	11 (7)	0 (0)
Hypophysitis	8 (5)	0 (0)
Blood lactate dehydrogenase increased	7 (4)	0 (0)
Nausea	5 (3)	0 (0)

## Discussion

In this retrospective analysis of uHCC, the combination of immunotherapy and targeted therapy demonstrates growing clinical promise. By analyzing 163 uHCC patients who received first-line treatment with tislelizumab plus lenvatinib, this study provides valuable real-world evidence on the efficacy and safety of this regimen, particularly within a large cohort predominantly consisting of HBV-related HCC patients. A key innovation of our work, compared to existing studies, lies in its larger real-world cohort and more detailed analysis of outcomes following conversion surgery, adding significant value to the current literature (Xu et al., 2024).

The observed ORR of 25.8% and DCR of 67.5% align with results reported in other ICI and TKI combination trials (Qin et al., 2023a). Notably, the median PFS of 13.87 months highlights the regimen’s capacity for durable disease control, surpassing the 6.8 months reported in the IMbrave150 trial (Finn et al., 2020b). However, caution is warranted in interpreting this result due to differences in study design, population characteristics, follow-up duration, and the immaturity of our OS data.

Encouragingly, 16.6% of patients achieved conversion to a resectable state through combination therapy and underwent surgical resection, providing a potential curative option for initially unresectable patients. These patients exhibited more favorable clinical features, including younger age, smaller tumor burden, and a lower incidence of PVTT. All surgical patients achieved R0 resection and a major pathological response, with a median RFS of 23.67 months. However, given the limited sample size of the conversion surgery subgroup (*n* = 27) and the relatively short follow-up period, these results should be interpreted cautiously, with long-term efficacy requiring further validation.

The role of local therapies, such as TACE, HAIC, and ablation, also deserves attention. Liver-directed therapy emerged as a protective factor for OS in both univariate and multivariate Cox analyses. Although interaction analyses with factors such as tumor burden did not reveal statistically significant differences, existing evidence strongly supports a synergistic effect between local and systemic therapies. Local treatments may enhance immunotherapy efficacy by inducing immunogenic cell death (Hao et al., 2024; Pinato et al., 2021), altering the tumor microenvironment (Tan et al., 2023; Wang et al., 2024b), and promoting the release of tumor antigens(Zhang et al., 2024a). Meanwhile, lenvatinib’s anti-angiogenic effects may improve tumor blood supply (Ladd et al., 2024), thereby enhancing drug delivery and the effectiveness of local treatments (Huang et al., 2020; Jain, 2005; Kano et al., 2009). This multi-modal treatment strategy provides a robust theoretical foundation for the combination therapy.

An important observation for discussion is that while tumor size ≥ five cm was an independent predictor of PFS, it did not correlate significantly with OS. Additionally, other high-risk factors, such as tumor number, PVTT, and extrahepatic metastasis, showed no significant differences in subgroup analyses. This suggests that the combination regimen may be effective across different risk profiles. However, in patients with a large tumor burden, while the regimen can delay disease progression, it may not ultimately improve OS. Biologically, lenvatinib inhibits angiogenesis and modulates the tumor immune microenvironment by blocking VEGF receptors. It normalizes tumor vasculature, alleviates hypoxia, and reduces the infiltration of immunosuppressive cells (Zhao et al., 2020). This primed microenvironment enhances the efficacy of tislelizumab, which can reactivate exhausted T-cells (Yi et al., 2022). This immunomodulatory effect may be particularly pronounced in HBV-related HCC, characterized by chronic inflammation, thereby contributing to delayed progression. However, in patients with extensive tumor burden, the inherent aggressiveness and immune escape mechanisms may limit the regimen’s ability to improve long-term survival outcomes.

Regarding safety, the combination therapy was well-tolerated, with a low incidence of high-grade toxicities. This favorable safety profile is particularly relevant in a real-world setting, as very few patients required dose adjustments. These findings support the use of this regimen as a viable first-line treatment.

This study has several limitations. First, the single-center, retrospective design introduces potential biases, such as patient selection bias, variations in treatment management, and outcome assessment bias. Second, the predominantly HBV-related cohort (84.7%) limits the generalizability of these findings to patients with HCC of other etiologies, such as hepatitis C virus (HCV) or non-alcoholic fatty liver disease (NAFLD), as well as to Western populations, where the underlying biology may differ. This should be considered a significant limitation rather than just a demographic characteristic. Third, the absence of data on HBV-specific factors, including viral load control and details of antiviral therapy, prevented an analysis of their impact on outcomes. These factors are crucial for clinical decision-making in HBV-endemic regions. Lastly, given the importance of tolerability and quality of life for first-line regimens, the lack of quality-of-life data or patient-reported outcomes is a considerable limitation that should be addressed in future studies.

Despite these limitations, this study provides valuable insights into the use of tislelizumab plus lenvatinib in real-world uHCC patients, particularly in the HBV-related population. Future multicenter studies are required to validate these findings and should incorporate comprehensive HBV-specific variables, patient-reported outcomes, and extended follow-up to fully assess the long-term value and applicability of this combination regimen in diverse populations.

## Conclusions

In this retrospective analysis of HBV-related uHCC, tislelizumab plus lenvatinib demonstrated favorable efficacy and safety. The regimen achieved a 25.8% ORR, 67.5% DCR, and a median PFS of 13.87 months, with 16.6% of patients undergoing successful conversion therapy. Tumor burden, particularly size ≥five cm, was associated with earlier progression but showed no significant correlation with OS. The low incidence of high-grade AEs supports its clinical applicability. These results may guide first-line therapeutic decision-making, especially in HBV-endemic regions, and further highlight the increasing relevance of immunotherapy-TKI combinations in managing uHCC.

## Supplemental Information

10.7717/peerj.20591/supp-1 Supplemental Information 1 Dataset

## Abbreviations

AEs: adverse events
AFP: alpha-fetoprotein
ANG2: angiopoietin-2
CI: confidence interval
CR: complete response
CT: computed tomography
CTCAE: Common Terminology Criteria for Adverse Events
DCR: disease control rate
FcγR: Fc gamma receptor
HAIC: hepatic arterial infusion chemotherapy
HBV: hepatitis B virus
HCC: hepatocellular carcinoma
HCV: hepatitis C virus
HR: hazard ratio
ICI: immune checkpoint inhibitor
MDT: multidisciplinary team
MRI: magnetic resonance imaging
mRECIST: modified Response Evaluation Criteria in Solid Tumors
NAFLD: non-alcoholic fatty liver disease
ORR: objective response rate
OS: overall survival
PD: progressive disease
PD-1: programmed cell death protein 1
PFS: progression-free survival
PR: partial response
PVTT: portal vein tumor thrombus
R0: microscopically margin-negative
RCTs: randomized controlled trials
RECIST: Response Evaluation Criteria in Solid Tumors
RFS: recurrence-free survival
SD: stable disease
TACE: transarterial chemoembolization
TKI: tyrosine kinase inhibitor
TRAEs: treatment-related adverse events
uHCC: unresectable hepatocellular carcinoma
VEGF: vascular endothelial growth factor

## Additional Information and Declarations

### Data Availability

The following information was supplied regarding data availability: The raw data are available in the Supplemental File.

## References

[ref-1] Bray F, Laversanne M, Sung H, Ferlay J, Siegel RL, Soerjomataram I, Jemal A (2024). Global cancer statistics 2022: GLOBOCAN estimates of incidence and mortality worldwide for 36 cancers in 185 countries. CA: A Cancer Journal for Clinicians.

[ref-2] Finn RS, Ikeda M, Zhu AX, Sung MW, Baron AD, Kudo M, Okusaka T, Kobayashi M, Kumada H, Kaneko S, Pracht M, Mamontov K, Meyer T, Kubota T, Dutcus CE, Saito K, Siegel AB, Dubrovsky L, Mody K, Llovet JM (2020a). Phase Ib study of lenvatinib plus pembrolizumab in patients with unresectable hepatocellular carcinoma. Journal of Clinical Oncology.

[ref-3] Finn RS, Qin S, Ikeda M, Galle PR, Ducreux M, Kim TY, Kudo M, Breder V, Merle P, Kaseb AO, Li D, Verret W, Xu DZ, Hernandez S, Liu J, Huang C, Mulla S, Wang Y, Lim HY, Zhu AX, Cheng AL (2020b). Atezolizumab plus Bevacizumab in unresectable hepatocellular carcinoma. New England Journal of Medicine.

[ref-4] Finn RS, Ryoo BY, Merle P, Kudo M, Bouattour M, Lim HY, Breder V, Edeline J, Chao Y, Ogasawara S, Yau T, Garrido M, Chan SL, Knox J, Daniele B, Ebbinghaus SW, Chen E, Siegel AB, Zhu AX, Cheng AL (2020c). Pembrolizumab as second-line therapy in patients with advanced hepatocellular carcinoma in KEYNOTE-240: a randomized, double-blind, phase III trial. Journal of Clinical Oncology.

[ref-5] Fukumura D, Kloepper J, Amoozgar Z, Duda DG, Jain RK (2018). Enhancing cancer immunotherapy using antiangiogenics: opportunities and challenges. Nature Reviews Clinical Oncology.

[ref-6] Hao Y, Zhu W, Li J, Lin R, Huang W, Ain QU, Liu K, Wei N, Cheng D, Wu Y, Lv W (2024). Sustained release hypoxia-activated prodrug-loaded BSA nanoparticles enhance transarterial chemoembolization against hepatocellular carcinoma. Journal of Controlled Release.

[ref-7] Huang A, Yang XR, Chung WY, Dennison AR, Zhou J (2020). Targeted therapy for hepatocellular carcinoma. Signal Transduction and Targeted Therapy.

[ref-8] Huinen ZR, Huijbers EJM, Van Beijnum JR, Nowak-Sliwinska P, Griffioen AW (2021). Anti-angiogenic agents-overcoming tumour endothelial cell anergy and improving immunotherapy outcomes. Nature Reviews Clinical Oncology.

[ref-9] Jain RK (2005). Normalization of tumor vasculature: an emerging concept in antiangiogenic therapy. Science.

[ref-10] Kano MR, Komuta Y, Iwata C, Oka M, Shirai YT, Morishita Y, Ouchi Y, Kataoka K, Miyazono K (2009). Comparison of the effects of the kinase inhibitors imatinib, sorafenib, and transforming growth factor-beta receptor inhibitor on extravasation of nanoparticles from neovasculature. Cancer Science.

[ref-11] Kudo M, Finn RS, Qin S, Han KH, Ikeda K, Piscaglia F, Baron A, Park JW, Han G, Jassem J, Blanc JF, Vogel A, Komov D, Evans TRJ, Lopez C, Dutcus C, Guo M, Saito K, Kraljevic S, Tamai T, Ren M, Cheng AL (2018). Lenvatinib *versus* sorafenib in first-line treatment of patients with unresectable hepatocellular carcinoma: a randomised phase 3 non-inferiority trial. Lancet.

[ref-12] Ladd AD, Duarte S, Sahin I, Zarrinpar A (2024). Mechanisms of drug resistance in HCC. Hepatology.

[ref-13] Llovet JM, Kudo M, Merle P, Meyer T, Qin S, Ikeda M, Xu R, Edeline J, Ryoo BY, Ren Z, Masi G, Kwiatkowski M, Lim HY, Kim JH, Breder V, Kumada H, Cheng AL, Galle PR, Kaneko S, Wang A, Mody K, Dutcus C, Dubrovsky L, Siegel AB, Finn RS (2023). Lenvatinib plus pembrolizumab *versus* lenvatinib plus placebo for advanced hepatocellular carcinoma (LEAP-002): a randomised, double-blind, phase 3 trial. The Lancet Oncology.

[ref-14] Llovet JM, Montal R, Sia D, Finn RS (2018). Molecular therapies and precision medicine for hepatocellular carcinoma. Nature Reviews Clinical Oncology.

[ref-15] Llovet JM, Ricci S, Mazzaferro V, Hilgard P, Gane E, Blanc JF, De Oliveira AC, Santoro A, Raoul JL, Forner A, Schwartz M, Porta C, Zeuzem S, Bolondi L, Greten TF, Galle PR, Seitz JF, Borbath I, Häussinger D, Giannaris T, Shan M, Moscovici M, Voliotis D, Bruix J (2008). Sorafenib in advanced hepatocellular carcinoma. New England Journal of Medicine.

[ref-16] Pinato DJ, Murray SM, Forner A, Kaneko T, Fessas P, Toniutto P, Mínguez B, Cacciato V, Avellini C, Diaz A, Boyton RJ, Altmann DM, Goldin RD, Akarca AU, Marafioti T, Mauri FA, Casagrande E, Grillo F, Giannini E, Bhoori S, Mazzaferro V (2021). Trans-arterial chemoembolization as a loco-regional inducer of immunogenic cell death in hepatocellular carcinoma: implications for immunotherapy. Journal for ImmunoTherapy of Cancer.

[ref-17] Qin S, Bi F, Gu S, Bai Y, Chen Z, Wang Z, Ying J, Lu Y, Meng Z, Pan H, Yang P, Zhang H, Chen X, Xu A, Cui C, Zhu B, Wu J, Xin X, Wang J, Shan J, Chen J, Zheng Z, Xu L, Wen X, You Z, Ren Z, Liu X, Qiu M, Wu L, Chen F (2021). Donafenib versus sorafenib in first-line treatment of unresectable or metastatic hepatocellular carcinoma: a randomized, open-label, parallel-controlled phase II-III trial. Journal of Clinical Oncology.

[ref-18] Qin S, Chan SL, Gu S, Bai Y, Ren Z, Lin X, Chen Z, Jia W, Jin Y, Guo Y, Hu X, Meng Z, Liang J, Cheng Y, Xiong J, Ren H, Yang F, Li W, Chen Y, Zeng Y, Sultanbaev A, Pazgan-Simon M, Pisetska M, Melisi D, Ponomarenko D, Osypchuk Y, Sinielnikov I, Yang TS, Liang X, Chen C, Wang L, Cheng AL, Kaseb A, Vogel A (2023a). Camrelizumab plus rivoceranib *versus* sorafenib as first-line therapy for unresectable hepatocellular carcinoma (CARES-310): a randomised, open-label, international phase 3 study. Lancet.

[ref-19] Qin S, Kudo M, Meyer T, Bai Y, Guo Y, Meng Z, Satoh T, Marino D, Assenat E, Li S, Chen Y, Boisserie F, Abdrashitov R, Finn RS, Vogel A, Zhu AX (2023b). Tislelizumab *vs* sorafenib as first-line treatment for unresectable hepatocellular carcinoma: a phase 3 randomized clinical trial. JAMA Oncology.

[ref-20] Ren Z, Ducreux M, Abou-Alfa GK, Merle P, Fang W, Edeline J, Li Z, Wu L, Assenat E, Hu S, Rimassa L, Zhang T, Blanc JF, Pan H, Ross P, Yen CJ, Tran A, Shao G, Bouattour M, Chen Y, Meyer T, Hou J, Tougeron D, Bai Y, Hou MM, Meng Z, Wu J, Li V, Chica-Duque S, Cheng AL (2023). Tislelizumab in patients with previously treated advanced hepatocellular carcinoma (RATIONALE-208): a multicenter, non-randomized, open-label, phase 2 trial. Liver Cancer.

[ref-21] Tan J, Fan W, Liu T, Zhu B, Liu Y, Wang S, Wu J, Liu J, Zou F, Wei J, Liu L, Zhang X, Zhuang J, Wang Y, Lin H, Huang X, Chen S, Kuang M, Li J (2023). TREM2(+) macrophages suppress CD8(+) T-cell infiltration after transarterial chemoembolisation in hepatocellular carcinoma. Journal of Hepatology.

[ref-22] Wang L, Cao J, Liu Z, Wu S, Liu Y, Liang R, Zhu R, Wang W, Li J, Sun Y (2024b). Enhanced interactions within microenvironment accelerates dismal prognosis in HBV-related HCC after TACE. Hepatology Communications.

[ref-23] Wang J, Sun L, Liu Y, Zhang Y (2024a). FIGNL1 promotes hepatocellular carcinoma formation *via* remodeling ECM-receptor interaction pathway mediated by HMMR. Current Gene Therapy.

[ref-24] Xu L, Chen J, Liu C, Song X, Zhang Y, Zhao H, Yan S, Jia W, Wu Z, Guo Y, Yang J, Gong W, Ma Y, Yang X, Gao Z, Zhang N, Zheng X, Li M, Su D, Chen M (2024). Efficacy and safety of tislelizumab plus lenvatinib as first-line treatment in patients with unresectable hepatocellular carcinoma: a multicenter, single-arm, phase 2 trial. BMC Medicine.

[ref-25] Yang C, Zhang H, Zhang L, Zhu AX, Bernards R, Qin W, Wang C (2023). Evolving therapeutic landscape of advanced hepatocellular carcinoma. Nature Reviews Gastroenterology & Hepatology.

[ref-26] Yau T, Park JW, Finn RS, Cheng AL, Mathurin P, Edeline J, Kudo M, Harding JJ, Merle P, Rosmorduc O, Wyrwicz L, Schott E, Choo SP, Kelley RK, Sieghart W, Assenat E, Zaucha R, Furuse J, Abou-Alfa GK, El-Khoueiry AB, Melero I, Begic D, Chen G, Neely J, Wisniewski T, Tschaika M, Sangro B (2022). Nivolumab *versus* sorafenib in advanced hepatocellular carcinoma (CheckMate 459): a randomised, multicentre, open-label, phase 3 trial. The Lancet Oncology.

[ref-27] Yi M, Zheng X, Niu M, Zhu S, Ge H, Wu K (2022). Combination strategies with PD-1/PD-L1 blockade: current advances and future directions. Molecular Cancer.

[ref-28] Zhang W, Chen X, Chen X, Li J, Wang H, Yan X, Zha H, Ma X, Zhao C, Su M, Hong L, Li P, Ling Y, Zhao W, Xia Y, Li B, Zheng T, Gu J (2024b). Fc-Fc interactions and immune inhibitory effects of IgG4: implications for anti-PD-1 immunotherapies. Journal for ImmunoTherapy of Cancer.

[ref-29] Zhang T, Song X, Xu L, Ma J, Zhang Y, Gong W, Zhang Y, Zhou X, Wang Z, Wang Y, Shi Y, Bai H, Liu N, Yang X, Cui X, Cao Y, Liu Q, Song J, Li Y, Tang Z, Guo M, Wang L, Li K (2018). The binding of an anti-PD-1 antibody to Fc*γ*RI has a profound impact on its biological functions. Cancer Immunology and Immunotherapy.

[ref-30] Zhang G, Xiao Y, Tan J, Liu H, Fan W, Li J (2024a). Elevated SLC1A5 associated with poor prognosis and therapeutic resistance to transarterial chemoembolization in hepatocellular carcinoma. Journal of Translational Medicine.

[ref-31] Zhao Y, Zhang YN, Wang KT, Chen L (2020). Lenvatinib for hepatocellular carcinoma: from preclinical mechanisms to anti-cancer therapy. Biochimica et Biophysica Acta (BBA)—Reviews on Cancer.

[ref-32] Zheng RS, Chen R, Han BF, Wang SM, Li L, Sun KX, Zeng HM, Wei WW, He J (2024). Cancer incidence and mortality in China, 2022. Zhonghua Zhong Liu Za Zhi.

[ref-33] Zheng RS, Zhang SW, Sun KX, Chen R, Wang SM, Li L, Zeng HM, Wei WW, He J (2023). Cancer statistics in China, 2016. Zhonghua Zhong Liu Za Zhi.

